# Squamous Cell Carcinoma in Patients with Inherited Epidermolysis Bullosa: Review of Current Literature

**DOI:** 10.3390/cells11081365

**Published:** 2022-04-17

**Authors:** Domenico Bonamonte, Angela Filoni, Aurora De Marco, Lucia Lospalluti, Eleonora Nacchiero, Valentina Ronghi, Anna Colagrande, Giuseppe Giudice, Gerardo Cazzato

**Affiliations:** 1 Section of Dermatology and Venereology, Department of Biomedical Sciences and Human Oncology (DIMO), University of Bari “Aldo Moro”, 70124 Bari, Italy; domenico.bonamonte@uniba.it (D.B.); angela.filoni@gmail.com (A.F.); aurorademarco94@gmail.com (A.D.M.); lucia.lospalluti@policlinico.ba.it (L.L.); 2 Unit of Dermatology and Venerology, Perrino Hospital, 72100 Brindisi, Italy; 3 Section of Plastic Surgery, Department of Emergency and Organ Transplantation (DETO), University of Bari “Aldo Moro”, 70124 Bari, Italy; eleonora.nacchiero@policlinico.ba.it (E.N.); valentina.ronghi@policlinico.ba.it (V.R.); giuseppe.giudice@uniba.it (G.G.); 4 Section of Pathology, Department of Emergency and Organ Transplantation (DETO), University of Bari “Aldo Moro”, 70124 Bari, Italy; anna.colagrande@gmail.com

**Keywords:** epidermolysis bullosa, squamous cell carcinoma, inflammation, genodermatosis, skin cancer

## Abstract

Epidermolysis bullosa (EB) is a group of rare congenital diseases caused by mutations in structural proteins of the dermal/epidermal junction that are characterized by extreme epithelial fragility, which determines the formation of bullae and erosions either spontaneously or after local mechanical traumas. In EB patients, skin fragility leads to many possible complications and comorbidities. One of the most feared complications is the development of cutaneous squamous cell carcinomas (SCCs) that particularly in the dystrophic recessive EB subtype can be extremely aggressive and often metastatic. SCCs in EB patients generally arise more often in the extremities, where chronic blisters and scars are generally located. SCCs represent a big therapeutic challenge in the EB population. No standard of care exists for the treatment of SCC in these patients, and therapy is based on small case studies. Moreover, the pathogenesis of cSCC in EB patients is still unclear. Many theories have been indeed postulated in order to explain why cSCC behaves so much more aggressively in EB patients compared to the general population. cSCC in EB seems to be the result of many complex interactions among cancer cells, skin microenvironment, susceptibility to DNA mutations and host immune response. In this review, we analyze the different pathogenetic mechanisms of cSCC in EB patients, as well as new therapies for this condition.

## 1. Introduction

### 1.1. Disease Definition and Epidemiology

Inherited epidermolysis bullosa (EB) is a complex clinical and pathological entity that includes over 30 phenotypically and/or genotypically distinct inherited diseases, all sharing an intrinsic common tendency toward mechanical skin fragility and bullae formation [1,2].

Based on cleavage level, four major types of inherited EB have been described: EB simplex (EBS), junctional EB (JEB), dystrophic EB (DEB), and Kindler syndrome (KS). Therefore, intraepidermal blistering is the most characteristic feature of EBS, while in JEB and DEB blisters arise from the *lamina lucida* and the *sub-lamina densa*, respectively. On the other hand, in KS, multiple cleavage planes have been described and may be present at the same time in the same patient [1,2].

Despite its large number of possible clinical variants, EB is still classified as a rare disease, as its prevalence in the USA accounts for 8.22 cases per million [3]. However, certain variants are generally more frequent than others, such as EBS, with a prevalence ranging from 6 to 30 cases per million, and DEB, with a prevalence of 6 cases per million in both the USA and Spain, and even 20 cases per million in Scotland [1,4].

### 1.2. Clinical Manifestations and Major Subtypes

Clinical presentation of EB is quite heterogeneous, as skin lesions may occur in different body sites with variable depth and extent, and other organs may be also involved with worse prognosis and severe complications [1,2].

As for EBS, its onset is usually at birth with possible delays for the localized forms. Skin is often the only involved organ, and blisters generally occur within the epidermal basal layer and thus usually do not lead to retracting scars and milia. The most common type of EBS is *localized EBS*, formerly known as *Weber–Cockayne*, where blisters occur exclusively on major trauma sites, such as hand palms and foot soles. On the other hand, the most common variant of generalized EBS is the *Dowling Meara* one, which is characterized by the presence of groups of intact small blisters, resembling herpetic lesions [2].

On the contrary, JEB includes two main clinical variants, known as *Herlitz JEB* and *non-Herlitz JEB*, which share the characteristic dental pitting and hypoplasia. *Herlitz JEB* is present at birth and involves all skin surfaces with the formation of exuberant granulation tissue, arising from skin erosions. Many other organs are usually involved, including the gastrointestinal tract, the upper airways and the genitourinary tract with profound growth retardation, severe anemia and a generally bad prognosis. On the other hand, even if *non-Herlitz JEB* is associated with a lower risk of upper airway involvement and occlusion, the mortality rate in these patients is only slightly lower than that registered in the *Herlitz* form [2].

As for DEB, it is possible to distinguish two genetic variants: dominant DEB (DDEB) and recessive DEB (RDEB), both characterized by deep cleavage planes within the skin, retracting scars, milia formation and possible esophageal involvement. However, the dominant form is generally less severe with a better prognosis compared to the recessive forms. RDEB includes three main subtypes: *severe generalized RDEB* (formerly known as *Hallopeau–Siemens*), *non-Hallopeau–Siemens RDEB* and *inverse RDEB. Severe generalized RDEB* is one of the most debilitating genetically transmitted diseases, with generalized blistering, retracting and even mutilating scars, profound growth retardation, multifactorial anemia, esophageal strictures and debilitating hand and foot deformities. Chronic renal failure and dilated cardiomyopathy may also occur, possibly leading to death [2].

In the end, KS is characterized by generalized blistering at birth and later development of poikilodermatous pigmentation and photosensitivity. Skin findings may include atrophic scarring and nail dystrophy, while extracutaneous complications may include severe colitis, esophagitis, urethral strictures and, rarely, ectropions [2].

Despite their clinical variability, all EB subtypes are characterized by a more or less increased risk of developing cutaneous squamous cell carcinoma (cSCC). Therefore, the present article aims to review the current literature in order to better understand the epidemiology and the pathogenesis of this feared and often lethal complication occurring among EB patients. 

## 2. Cutaneous Squamous Cell Carcinoma in Epidermolysis Bullosa

### 2.1. Epidemiology

The risk for cSCC among EB subtypes seems to reflect the severity of the disease itself. Therefore, cSCC occurs very rarely among EBS patients (2.6%) [5], and according to some authors, EBS is not actually related to any higher risk compared to the general population [6]. On the contrary, higher percentages are indeed registered among KS, JEB, DDEB and RDEB patients. KS and DDEB seem to display a similar risk for cSCC, with a prevalence of 6% for both subtypes [5]. JEB and RDEB, on the other hand, are the subtypes more frequently related to the development of cSCC, with variable percentages ranging from 6.8% to 16.2% and from 35.4% to 70%, respectively [5,7,8].

Unlike the general population, cSCC in EB usually displays a peculiar aggressive clinical behavior [1]. It generally occurs at younger ages, with a mean age of onset ranging from 32.8 to 36 years old among all EB subtypes, with even earlier peaks of incidence among RDEB patients (29.5 years old) [5,7].

Among all subtypes, RDEB is associated not only with the highest incidence of cSCC and the youngest onset, but also with the worst prognosis [9]. The cumulative risk for cSCC in RDEB patients is indeed dramatically high, with possible differences according to patients’ age (7.5% by the age of 20 and over 90.1% by the age of 55) [10] and disease severity (10% by the age of 35 among RDEB patients with and intermediate generalized disease and at least 76.1% among RDEB patients with a severe generalized form) [8]. Therefore, the cumulative risk of death from cSCC in RDEB patients seems as well to reflect patients’ age, as it is 57.2% by the age of 35 and 87.3% by the age of 45 [10].

### 2.2. Clinical Presentation

In EB patients, cSCC preferably occurs on chronic non-healing ulcers located on bony prominences [1,8,11]. Therefore, early diagnosis of SCC can be very difficult since it can present similarly to typical chronic ulceration with scarring and crusting; similarly to burn scar tumors, SCCs in EB patients usually start as an ulcer margin [1,5,11].

Among all EB subtypes, limbs are the most commonly affected sites, with a consistent involvement of both lower (54.7%) and upper (30.8%) extremities which becomes prominent in RDEB, accounting for the 91.3–95% of cases [5,7,12]. On the other hand, mucosal SCC rarely occurs in EB, with a mean incidence of 8.6% among all variants [5].

At first diagnosis, ulceration is the most common clinical feature among all EB subtypes (44.9%), with a macroscopic diameter more frequently larger than 2 cm (59.1%) in all groups [5]. RDEB patients demonstrate similar percentages with ulceration and diameter larger than 2 cm in 30.4% of cases [12].

Due to its clinical aggressive behavior, in EB patients, cSCC usually presents with multifocal lesions at diagnosis (63.6%) with an average of three tumors per patient in RDEB and two tumors per patient in both JEB and DDEB [7].

Local recurrences generally occur from 12 to 14.9 months after the first surgery with an incidence ranging from 18.2% to 36.1%, while metastatic disease usually develops in 36.1–38.7% of cases, thus leading to death (Table 1) [5,7].

### 2.3. Histological Findings

Despite its aggressive clinical behavior, cSCC in EB patients presents as histologically well-differentiated in most cases (55.4–73.9%) [5,7,12]. Interestingly, in RDEB, the percentage of well-differentiated cases seems to be even higher, reaching peaks of 91.4% in localized disease and of 85% in metastatic disease [5].

However, over time, 36% of EB patients may display a certain tendency to shift from well-differentiated forms to poorly differentiated ones, thus leading to a progressive worsening of prognosis [7]. These cSCCs may chronically involve any area of the skin, esophagus and mouth [7]. While the diagnosis in poorly differentiated forms of cSCC is quite simple from a dermatopathological point of view, there is some difficulty in the differential diagnosis between well-differentiated forms of cSCC and pseudoepitheliomatous hyperplasia [7,13]. In fact, in the skin of subjects affected by EB, there is a continuous alteration which leads to a certain difficulty in differentiating these different conditions on a morphological basis.

### 2.4. Pathogenesis

The pathogenesis of cSCC in EB patients is still unclear. Many theories have been indeed postulated in order to explain why cSCC behaves so much more aggressively in EB patients compared to the general population. Genetic and epigenetic factors seem to be involved, as cSCC in EB seems to be the result of many complex interactions among cancer cells, skin microenvironment, susceptibility to DNA mutations and host immune response [1,14,15]. Given the prevalence of cSCC in patients with RDEB, most of the studies on the pathogenesis and molecular aspects of these tumors have been conducted in this subgroup of patients.

#### 2.4.1. Genetic Factors and UV Damage

The exact role of UV exposure in the development of EB-cSCC is still somewhat controversial. While on one hand it has been indeed demonstrated that UV exposure plays only a marginal role in EB-cSCC compared to the general population, on the other hand, in KS, UV-induced skin damage may contribute to tumor onset and progression, as loss of kindlin-1 seems to increase the release of reactive oxygen species, thus sensitizing keratinocytes to solar damage [1].

However, despite sharing similar genetic profiles with UV-induced cSCC, EB-cSCC displays certain peculiar genetic features which can possibly explain the distinct clinical behavior of these two forms of SCC. First of all, p53 mutations in RDEB-cSCC are more similar to those normally found in other types of cancer such as lung cancer than to those found in UV-cSCC [14]. Furthermore, UV does not induce driver mutations in RDEB-cSCC, as they are generated endogenously by higher activity rates of the APOBEC (apolipoprotein B mRNA editing enzyme, catalytic polypeptide-like) enzyme family [1,16]. Interestingly, in RDEB-cSCC, the highest activity rates of the APOBEC enzyme family are indeed registered on chronic wounds, thus possibly explaining why cSCC preferably occurs in these areas rather than on UV-exposed ones [1,16].

On the other hand, KS-cSCC seems to represent once more a distinct entity, as it displays different molecular signatures compared not only to UV-cSCC in the general population, but also to RDEB-cSCC [1]. Therefore, different genetic profiles may also justify the premature senescent features of keratinocytes isolated from KS-cSCC, thus underlying new possible differences compared to other EB-cSCC [1].

As for JEB, a peculiar genetic finding has been recently demonstrated, as cases of over 100 primary cSCCs due to altered laminin-332 function have been isolated. Laminin-332 is indeed an extracellular matrix (ECM) component of the epithelial basement membrane which has been historically associated with the development of JEB, as its expression may induce generalized intermediate JEB when reduced or altered. However, laminin-332 not only plays an essential role in the development of JEB, but also is highly overexpressed in many types of epithelial tumors, also representing a crucial step in many different cSCC-promoting pathways. For these reasons, recent findings seem to have isolated specific lamin-332 mutations in JEB patients which can provide lamin-332 with tumorigenic properties, thus increasing the risk for cSCC in JEB [17].

#### 2.4.2. The Role of the Microenvironment

Although genetics plays a crucial role in tumorigenesis, the crosstalk between cancer cells and their microenvironment may certainly influence disease progression and clinical outcome as favorable local conditions may justify cancer invasion and distant spreading. 

In EB-cSCC, the microenvironment seems to play an even more important role compared to other types of cancer, as tumorigenesis in EB patients is deeply related to the impaired wound healing process, local inflammation and ECM inconsistency, especially in RDEB [1,15].

#### 2.4.3. Altered Wound Healing Process and Fibrosis

Wound healing is a complex and delicate process aimed at re-establishing tissue integrity after injury. It usually involves three phases known as “inflammation”, “new tissue formation” and “remodeling”. Dermal fibroblasts and myofibroblasts play a crucial role in the second phase. Their activation and regulation depend on ECM composition and interaction with other cell types. This delicate phase is strongly impaired in EB patients, especially among RDEB ones, where impaired wound healing clinically leads to a delayed healing process, exuberant fibrosis, retracting scars and ultimately cancer. 

As UVs do not have such a central role in the pathogenesis of cSCC in EB patients compared to patients without EB, endogenous factors, such as impaired wound healing, seem to play a pivotal role in tumorigenesis, thus possibly explaining why cSCC in these patients preferably occurs on chronic non-healing wounds rather than on sun-exposed areas [1,18].

As for RDEB, it became the perfect model for molecular studies regarding cSCC pathogenesis and aggressive clinical behavior. RDEB is indeed genetically determined by a genetic deficiency of the COL7A1 gene, thus causing a clinically relevant absence of type VII collagen (C7) within the skin [1]. Therefore, in RDEB patients, the genetically determined loss of C7 directly interferes with wound healing. It has been demonstrated indeed that C7 loss enhances keratinocyte migration and invasion, reduces epithelial differentiation, promotes epithelial–mesenchymal transition and upregulates tumorigenesis and angiogenesis through TGF-β1 signaling [1,15,19,20].

However, the role of C7 in RDEB tumorigenesis is still controversial. Retroviral transduction of C7 into RDEB patients’ keratinocytes seems to increase cancer cell migration and invasion, although laboratory techniques to restore C7 presence on RDEB skin may lead to excessive C7 concentrations, thus not reflecting physiological conditions and possibly influencing the reliability of results [21]. On the other hand, some authors demonstrated that cSCCs from RDEB patients express variable percentages of C7, thus suggesting that cSCC in RDEB may arise regardless of C7 skin concentrations [22]. According to this theory, further investigations seem to suggest that the only tumorigenic domain of C7 is the so-called “N-terminal non-collagenous domain” (NC1); therefore, only C7 expressing this domain can actually drive cancer transition, unrelatedly to its concentration on patients’ skin [23].

Besides C7 deficiency in RDEB patients, fibrosis due to the altered healing process in all EB patients leads to the formation of a permissive cancer environment, with a progressive fibroblast conversion into carcinoma-associated fibroblasts (CAFs). Indeed, CAFs in both EB cancer and non-cancer skin contribute to microenvironment alteration through the release of multiple cytokines and chemokines, thus also leading to inflammation [1,24]. 

TGF-β is a crucial regulator of fibroblast differentiation and fibrotic response. For these reasons, it is not surprising that TGF-β also displays an important role in tumorigenesis. TGF-β may indeed promote keratinocyte dedifferentiation, thus facilitating cancer transition [15]. In RDEB patients, the upregulation of TGF-β signaling seems to be related to C7 depletion, as both pathways are extremely interconnected [15]. When C7 is deficient, TGF-β expression is increased, thus leading to an enhanced collagen 1 release and a thicker dermis [15]. Moreover, a stiffer cancer stroma may drive tumor progression through a mechanosensing signaling mediated by β1 integrin, activated focal adhesion kinase (FAK) and phosphoinositide 3-kinase (PI3K) [15]. Interestingly, TGF-β genes are overexpressed in both cancer and non-cancer RDEB skin, thus indicating once more that RDEB skin intrinsically offers a favorable environment for cancer [25].

However, as for C7, the role of TGF-β in EB tumorigenesis is still controversial, as certain authors seem to recognize different possible TGF-β-mediated pathways which can at least partially justify different cellular responses to TGF-β. The activation of the canonical TGF-β pathway through the addition of exogenous TGF-β to cancer RDEB keratinocytes seems to arrest cellular proliferation, while the inhibition of the same pathway at an intracellular level drives different and apparently controversial cellular responses, thus suggesting the existence of an intricate intracellular network of pathways regulated by TGF-β that we may not yet completely understand [26].

However, tumor progression in EB patients still represents a complex event where many other biological markers are involved. Metalloproteinases (MMPs) are a family of endopeptidases that are able to actively cleave ECM components, thus facilitating tumor progression and spreading [27]. MMP-7 and MMP-13 are overexpressed in EB-cSCC, and MMP-7 also seems to promote cellular proliferation through the shedding of the heparin-binding epidermal growth factor-like growth factor and the subsequent activation of the epidermal growth factor receptor (EGFR) [28].

#### 2.4.4. Inflammation and Local Immune Response

Inflammation is a well-known cancer-promoting factor in both EB and non-EB patients. In particular, recent studies have demonstrated that serum IL-6 levels in RDEB patients are higher compared to those of healthy controls and correlate to disease severity and extent [1]. In RDEB patients, IL-6 may indeed promote cancer progression through both fibrosis and CAF activation, thus providing an interesting link between systemic inflammation and cancer development [1]. 

However, despite a certain degree of systemic inflammation, both generalized and skin-localized immune deficiencies are likely to occur in RDEB patients [29].

Interestingly, recent works have demonstrated a reduced inflammatory infiltrate in RDEB-cSCC compared to non-RDEB-cSCC. In particular, a significant reduction in CD3+, CD4+ and CD68+ has been demonstrated in RDEB-cSCC compared to primary cSCC in patients without EB, with a further significant reduction in CD3+, CD4+, CD8+ and CD20+ compared to secondary cSCC (postburns and postradiotherapy), thus suggesting that a certain degree of immune tolerance towards cancer antigens is present within RDEB skin [29]. Moreover, reduced lymphocytic peritumoral infiltrate is related to high mobility group box 1 (HMGB1) overexpression within cSCC both in non-EB and EB patients [29]. This aspect seems to relate inflammation to local immune deficiency in cSCC, as HMGB1 displays a pro-inflammatory cytokine-like function, while a reduced lymphocytic infiltrate causes immune tolerance and promotes cancer progression [30].

#### 2.4.5. Superinfections

Microbial skin superinfections in EB patients seem to contribute to cancer development in many ways. First of all, they can promote skin inflammation, thus enhancing local immune dysregulation [1]. Furthermore, infections are well-known risk factors for wound-healing failure, and microbial agents on EB skin may worsen an already impaired process and promote cancer in chronic non-healing wounds [1].

Therefore, an intrinsic major susceptibility to skin *Staphylococcus aureus* infections has been demonstrated on RDEB skin, regardless of its integrity [1]. C7 deficiency may indeed destabilize the ECM in lymphoid conduits of the spleen and lymph nodes, thus facilitating bacterial infections [1]. On the other hand, human papillomavirus (HPV) infections do not seem to be related to cSCC in RDEB patients, despite being well-known causative agents of cutaneous and mucosal SCC in the general population [1].

### 2.5. Biomarkers

Several molecules have been proposed as possible biomarkers for the development of cSCC in RDEB patients. Interestingly, cancer-type SLCO1B3, encoding for a family of anion-transporting polypeptides, has been isolated selectively in RDEB-cSCC cells, and its levels on liquid biopsies of tumor-bearing mice were higher than those of healthy subjects, thus displaying an interesting potential role as a biomarker for cSCC in RDEB patients [31].

Moreover, further studies focused on possible biomarkers for disease progression and described how serine proteases C_1r_ and C_1s_ are significantly overexpressed in advanced cSCC from RDEB patients, suggesting their potential for use not only in predicting disease progression but also as potential targets for new therapeutic approaches in metastatic cSCC [32].

### 2.6. Management

#### 2.6.1. Clinical Evaluation

Patients at highest risk for cSCC, such as RDEB patients with severe generalized forms, should undergo a full skin examination every 3–6 months from age 10, with shorter follow-ups (every 3 months at maximum) for patients with an already positive history for cSCC. Therefore, the clinical examination should be performed by qualified medical personnel and should include all body areas. However, particular attention should be given to specific high-risk areas, such as non-healing wounds (lasting more than 4 weeks), wounds with exuberant granulation tissue, deep ulcers with infiltrated margins, persistent hyperkeratotic areas and/or erosions. 

Since these carcinomas can be very difficult to identify, dermoscopy, a non-invasive tool, can help in the suspicion of cSCC. The poorly differentiated cSCC forms are characterized by a red predominant color and dotted or irregular vessels; on the contrary, the well-differentiated cSCC shows a white color, with white perifollicular circles, perivascular halos and a polymorphous vessel patter [33]. If necessary, multiple biopsies of suspicious lesions should be performed for histological examination [6].

#### 2.6.2. Primary Tumor

All EB patients diagnosed with cSCC should be managed by a multidisciplinary team, involving many medical specialists, such as dermatologists, plastic surgeons, histopathologists and oncologists. Furthermore, patients with infiltrated or large cSCCs (>5 cm) may benefit from imaging techniques in order to assess their surgical accessibility and the eventual involvement of underlying structures [6].

#### 2.6.3. Regional Lymph Node

In case of involved lymph nodes at diagnosis (during clinical or echographic examination), ultrasound-guided FNA should be performed; if negative, no further exams are required besides clinical and ultrasound follow-up every 3 months. On the other hand, in the case of lymph node positivity for cSCC metastasis, regional lymph node clearance could be taken into consideration, although there is no current evidence that this can actually affect prognosis [6].

#### 2.6.4. Distant Metastasis

Patients with a primary cSCC larger than 5 cm in maximum diameter or those with symptoms suggestive of metastatic spread should undergo staging, possibly with FDG-PET and CT combined [6].

### 2.7. Treatment

The first-line therapy for the majority of EB-cSCCs is surgical excision. Ideally, the tumor should be surrounded by a 2 cm excision margin, although this is often difficult to perform in clinical practice, especially in RDEB patients. Interestingly, although many minimally invasive surgical techniques have been proposed and used in RDEB-cSCCs, there is no clear evidence of their superiority over classical wide surgical excision [6]. Moreover, in order to improve surgery recovery, some authors suggest that dermal substitutes may be placed after surgery with better outcomes than simple second-intention wound closure [34]. Whilst many different treatment modalities have been tried in EB cancers, some patients chose to avoid tumor clearance surgery by limb amputation in favor of living with their SCC to maintain independence. This highlights the need to balance patient post-intervention quality of life and cancer treatments.

Electrochemotherapy, a local treatment that combines low-dose intralesional or systemic cytotoxic drugs (bleomycin or cisplatin) and the application of high-intensity electric pulses, has been described as a potential treatment in eight patients with favorable results [35,36].

Radiotherapy has also been widely used for both definitive and palliative treatment, as well as to reduce original tumor size, thus facilitating radical surgical excision [6].

Adjunctive topical therapies may be considered in EB-SCC. Topical 5% imiquimod has been indeed successfully used in a few cases of in situ EB-cSCC, although results are still conflicting and not always promising [7]. On the other hand, PDT has been successfully used in a few other cases of in situ EB-cSCC, although it was poorly tolerated [37].

Oral retinoids demonstrated a certain efficacy in the chemoprevention of SCCs in the organ transplant population [38], although no effects have been demonstrated yet in EB [1,39].

Local recurrences and/or distant metastasis may require other treatments, possibly systemic.

Conventional chemotherapy is not commonly used to treat metastatic cSCC in RDEB patients. Agents used in the literature include cisplatin, carboplatin and fluorouracil, although they often result in poor responses, supporting recommendations that risks may outweigh potential benefits [6].

On the other hand, more and more reports seem to suggest the use of target drugs in metastatic disease, suggesting that cetuximab (an epidermal growth factor receptor (EGFR) inhibitor) may be useful in the treatment of advanced cSCC [6,7,8,9,10,11,12,14,15,16,17,18,19,20,21,22,23,24,25,26,27,28,29,30,31,32,33,34,35,36,37,38,39,40]. RDEB-cSCC, indeed, frequently overexpresses EGFR, although a certain variability among patients has been described and may influence clinical responsiveness. However, reports published so far seem to suggest that cetuximab efficacy in metastatic EB-cSCC is higher when it is administered early, thus leading to better chances of survival [41]. Moreover, also due to its overall clinical safety, an association between cetuximab and immune-checkpoint inhibitors has also been suggested [38].

After the approval of cemiplimab (a PD-1 inhibitor) for the treatment of patients with metastatic or locally advanced SCCs who cannot undergo curative surgery or radiation [38], some authors have reported the successful use of this programmed-cell death receptor (PD-1) inhibitor in RDEB-cSCCs [7,42,43].

Furthermore, a prospective, multi-center, phase II trial (Eudra CT-No. 2016-002811-16) is currently evaluating the administration of nivolumab (an anti-PD1 monoclonal antibody) for the palliative treatment of DEB patients with locally advanced or metastatic cSCC, unresponsive to other systemic therapies [40], although a few studies have reported that PD-L1 expression is predictive of clinical outcome in cSCC, so further investigations are still necessary [42,43].

Moreover, following in vitro work in EB-cSCC cell lines and in vivo mice models, a clinical trial is currently underway in order to assess rigosertib (a tumor-specific PI-3K and polo-like kinase inhibitor) efficacy in advanced SCC non-responsive to standard treatments (ClinicalTrials.gov: NCT01807546) [13,44]. Other recent preclinical studies have also identified the JAK1/2 inhibitor ruxolitinib [45] and TGF-ßR1 kinase inhibitors [26] as potential new therapies for RDEB-associated SCCs. 

## 3. Conclusions

Patients with RDEB constitute a subset of patients more likely to develop primary malignant skin lesions, including the dreaded cSCC. In recent years, progress has been made in order to improve the outcome and the prognosis quoad vitam et valetudinem of these patients, although much still remains to be explored and resolved, starting from the evidence that a reduced immunotolerance and immunosurveillance is the molecular basis for the onset and progression of such neoplasms.

## Figures and Tables

**Table 1:** Main features of cSCC in EB patients.

Age of onset	32.8–36 years old
Involved sites	Upper and lower limbs and extremities
Number of lesions at first diagnosis	3 tumours per patient in RDEB and 2 tumours per patient in both JEB and DDEB
Main clinical features	Ulceration
Size at diagnosis	>2 cm (maximum diameter)
Histological differentiation	Well differentiated
Time to local recurrence	12–14.9 months after first surgery
Risk of developing distant metastasis	36.1–38.7%
